# Disseminated cutaneous coccidioidomycosis masquerading as acne keloidalis nuchae

**DOI:** 10.1016/j.jdcr.2023.06.047

**Published:** 2023-07-17

**Authors:** Iesha Ticknor, John Jay Cadavona, Keith D. Roby, David G. Cotter

**Affiliations:** a Kaiser Permanente Bernard J. Tyson School of Medicine, Pasadena, California; b Department of Medicine, David Geffen School of Medicine at UCLA, Los Angeles, California; c Department of Dermatology, University of California, San Diego, San Diego, California; d Department of Dermatology, Southern California Permanente Medical Group, San Diego, California; e Kirk Kekorian School of Medicine at UNLV, Las Vegas, Nevada; f Las Vegas Dermatology, Las Vegas, Nevada

**Keywords:** acne keloidalis nuchae, *Coccidioides immitus*, cutaneous coccidioidomycosis, fungal infection, valley fever

## Introduction

Coccidioidomycosis, also known as Valley fever or desert rheumatism, is a fungal infection endemic to the dry, arid environments of the Southwestern United States and Northern Mexico. Infection is most often the result of inhalation of airborne arthroconidia from the environment. In healthy individuals who become infected with coccidioidomycosis, 60% of cases are asymptomatic. Approximately 40% of cases will experience a mild to moderate but self-resolving flu-like infection.^1^ Of those with symptomatic infection, extrapulmonary dissemination is rare, and most often occurs in the setting of immunosuppression, pregnancy, and in certain racial/ethnic groups.^1^ In a retrospective analysis by Adam et al,^2^ the skin was the most common site of dissemination.^2^ Cutaneous manifestations of *Coccidioides* infection occur through the following 3 mechanisms: reactive eruptions, primary inoculation, and hematogenous dissemination. Extrathoracic reactive skin eruptions are noninfectious immune-mediated reactions to the primary pulmonary infection. Primary cutaneous infection is exceedingly rare and results from direct inoculation of organisms from cutaneous trauma.^1^ The diagnosis of disseminated cutaneous coccidioidomycosis can be challenging given the morphologic heterogeneity. Cutaneous lesions are nonspecific and have been described to masquerade as several distinct infectious, inflammatory, and neoplastic skin conditions. Here, we present an unusual case of disseminated cutaneous coccidioidomycosis mimicking acne keloidalis nuchae.

## Case report

An immunocompetent and previously healthy 52-year-old African American man with a history of atopic dermatitis presented for evaluation of a persistent pruritic eruption on his posterior aspects of the scalp and neck of 9 months duration. Before our evaluation, he had been treated for both atopic dermatitis and lichen simplex chronicus with topical and intralesional steroids without significant improvement. Several months prior to his skin eruption, he was seen in his local emergency department for fever, chills, fatigue, cough, dyspnea, intermittent hemoptysis, and a headache. His history was also notable for a 20-pound unintentional weight loss. A chest radiograph at the time revealed a right lower lobe consolidation, and he was subsequently treated with antibiotics for presumptive bacterial pneumonia.

**Fig 1:**
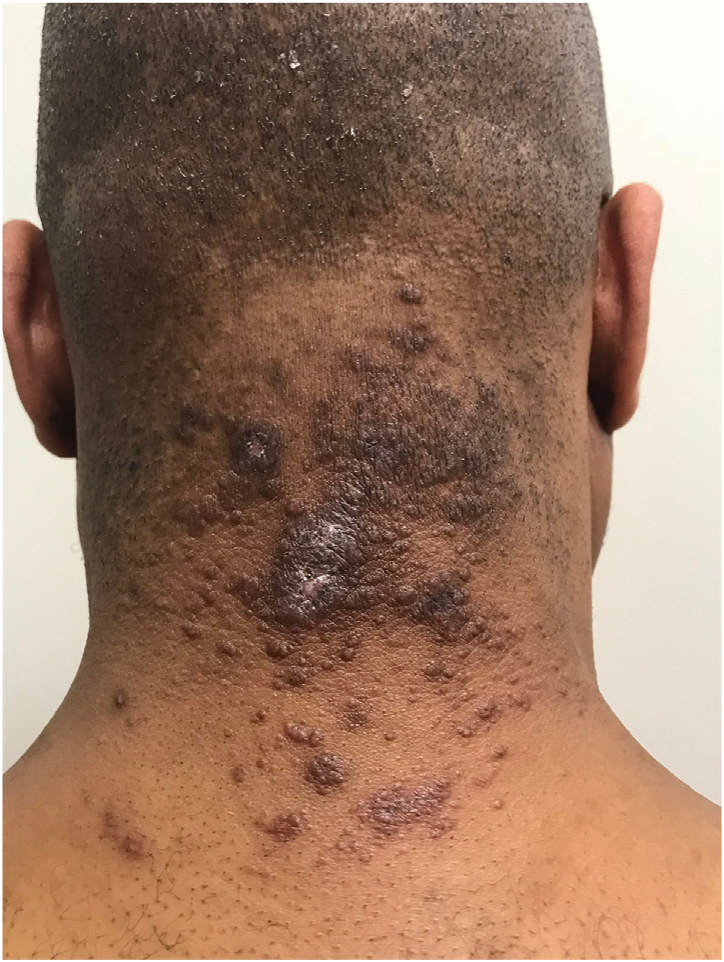
Clinical photographs at initial presentation. Erythematous papules coalescing into violaceous plaques on the posterior aspects of the scalp and neck.

**Fig 2:**
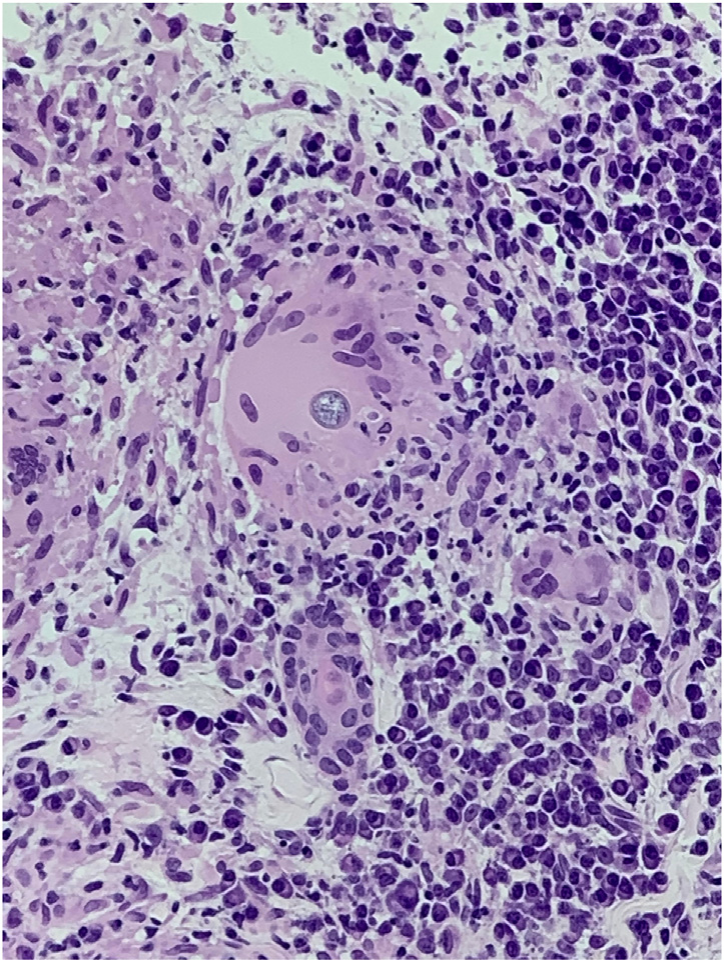
Spherule containing multiple anucleate endospores (*black arrow*) within a multinucleate giant cell, characteristic of coccidioidomycosis infection (hematoxylin-eosin stain, original magnification: 10×).

On our initial examination, erythematous papules coalescing into violaceous plaques on the posterior aspect of the scalp and nape of the neck were noted (Fig 1). Clinically, our differential diagnosis included acne keloidalis nuchae given the distribution of the papular eruption; however, pustules were not seen and lesions extended down the neck, beyond the hair line. A skin biopsy from the posterior aspect of the neck demonstrated granulomatous inflammation with giant cell reaction and the presence of thick-walled spherules with anucleate endospores (Fig 2), consistent with cutaneous coccidiomycosis. A separate representative tissue sample sent for culture subsequently grew *Coccidioides immitis*, confirming the diagnosis. Susceptibility testing against antifungal drugs was not performed by the laboratory. His initial Coccidioides antibody titer by complement fixation was 1:32. He was treated with systemic fluconazole without improvement in serologic titers, skin disease, or pulmonary symptoms. He was switched to posaconazole, which led to clinical improvement and decrease in Coccidioides titer to 1:8. After several weeks, he developed hypertension, hypokalemia, and headaches and was diagnosed with posaconazole-induced pseudohyperaldosteronism. He was treated with triamterene, a potassium-sparing diuretic, switched to isavuconazole, and his blood pressure and renal function subsequently normalized. Coccidioides antibody titers decreased to 1:4 and at the last follow up, skin involvement had significantly improved as well.

## Discussion

Coccidioidomycosis is a fungal infection caused by inhalation of *Coccidioides immitis or Coccidioides posadasii* spores from disturbed soil or dust. Although traditionally occurring in the hot, arid regions of the Southwestern United States and Central America, cases have recently been reported as far north as Washington state in patients without a history of travel to endemic areas.^3^ Due to increasing temperatures and shifting precipitation patterns, models predict that the endemic areas will continue to swell.^4^ The risk of primary pulmonary infection is greatest in those exposed to dust or soil in endemic areas, often as a result of occupational or recreational outdoor activities. Most cases of coccidioidomycosis are asymptomatic in healthy individuals; however, patients with impaired cell-mediated immunity and certain ethnic groups, such as African Americans and Filipinos, are at an increased risk of severe symptomatic infection. Cough, dyspnea, fever, night sweats, and fatigue are systemic symptoms commonly associated with severe infection. Disseminated infection occurs in <1% of cases, with the skin being one of the most common extrathoracic sites; however, dissemination may involve virtually any organ system.^1^ Cutaneous coccidioidomycosis, such as syphilis and tuberculosis, has been termed a “great imitator,”^5^ given the myriad of morphologies it can present with and its ability to evade diagnosis by even the most astute clinicians. Dermatologic manifestations of coccidioidomycosis are incredibly diverse and morphologies include erythematous papules, pustules, abscesses, nodules, ulcers, papules, and plaques.^6^ The erythema seen in lighter skin types may not be as easily identifiable in skin of color, further obfuscating the diagnosis. Atypical cutaneous presentations have been described to mimic several infectious, inflammatory, and neoplastic skin diseases, including leprosy,^7^ cicatricial alopecia,^8^ lupus pernio,^9^ and mycosis fungoides.^10^ Our report highlights an extremely rare case of disseminated cutaneous coccidioidomycosis in a patient presenting with erythematous papules and plaques on the occipital scalp mimicking acne keloidalis nuchae.

The expanding geographic range of the organism in addition to the incredible clinical variability of coccidioidomycosis poses a significant diagnostic challenge, and as in our case, may lead to delays in accurate diagnosis and initiation of appropriate antifungal treatment. Systemic triazole antifungal medications, such as fluconazole or itraconazole, are the first-line treatment of choice for patients with nonmeningeal-disseminated coccidioidal soft tissue infection. As in this case, posaconazole, isavuconazole, and voriconazole have been used as second-line therapy. Liposomal amphotericin B is usually reserved for treatment of severe, rapidly progressive, or refractory cases because of its increased toxicity compared with azoles.

Physicians should consider *Coccidioides* infection in the differential diagnosis for patients presenting with atypical skin lesions with a history of intractable pulmonary symptoms, especially in ethnic groups at increased risk of severe disease, those with impaired cell-mediated immunity, and those residing in endemic areas.

## Conflicts of interest

Dr Cotter is a consultant for Abbvie, Galderma, Sanofi, Regeneron, Castle, DermTech, Journey, Bristol Myers Squibb, Arcutis, Boehringer Ingelheim. Author Ticknor, Drs Cadavona and Roby have no conflicts of interest to declare.
